# Supplementary material for: Nutritional assessment and associated factors in children with congenital heart disease—Ethiopia
Source: PLoS One. 2022 Sep 23;17(9):e0269518. doi: 10.1371/journal.pone.0269518 (PMC9506638; doi:10.1371/journal.pone.0269518)
Supplement: Attachment — Submitted filename: Response to Reviewers.docx [file pone.0269518.s002.docx]

**Response to Reviewers**

Dear Reviewers, we would like to thank you very much for your precious time in revising the paper and for your invaluable comments to enrich the manuscript.

We would like apologies as well for our delayed responses as we have postponed the date for our response after informing the editorial team as we have been doing another research which is a continuation of this one that is assessing the impact of corrective intervention in malnourished children with CHD.

My responses and corrections are as per the comments forwarded from reviewer #2.

Reviewer#2: 
1- The current manuscript is written in a class education fashion and this fashion could not be accepted as journal paper. You should rewrite the manuscript and follow carefully the journal's guidelines for authors regarding proper manuscript structure and design.

**Response**: I have revised the overall design of the manuscript according to journal's guidelines for authors. The changes are attached to separately as “**Revised Manuscript with Track Changes**”.

2- The authors enrolled children from 1 year to 17year old. Assessing nutritional status in a 2 year old child is not like assessing nutritional status in a 17 year old teenage. The age limit should be re considered.

**Response:** We have revised the age group as per your comment (see comment #3)…**page 16.**

Of course the nutritional assessment will be different for different age groups and we used WHO standard growth curves accordingly and especially for older children we used WHO Body mass Index curves.

3- The authors should make a separate and complete analyses using subgroup analyses for different age groups (i.e., <5 yr, from 5 to 12 yr, from 12 to 17 yr).

**Response:** Our research has tried to enroll all pediatric age groups which is by definition those less than 18 years of age. The age range in our research is from 3 months up to 17 years.

As per your comment we have recategorized the age group into those less than 1 year (infants), 1 to 3 year (toddler), 3 to 5 year (preschool), 5 to 10 year and those above 10 years of age (which is adolescent age group according to WHO definition).(**page 16)**

For assessment of Weight for age in those less than 10 years we used WHO child curve standards while for those above 10 years we used WHO Body Mass Index curve. There were only two patients whose age is above ten year and one is found to be underweight while the other one is normal which makes difficult to reach into a conclusion because of few number of patients (I have added an explanation under “**limitation of the study”). (page 22)**

In the result, I have revised a sentence which describes the occurrence of underweight in those above five years in comparison with those between the age of 3 and 5 year. **(page 18)**

4- A comparison group composed of healthy children from the same region, same community and same age is needed. This is very important to measure properly the significance of changes in nutritionalstatus.

**Response**: Of course, it would have been nice to do a comparative study, but our patients are coming from all over the country as the cardiac center is the only one located in the capital city. So it will be difficult to construct another group of children with similar backgrounds.

5- In the methodology and study protocol, calculate the minimal sample size needed to conduct the study.

**Response:** 228 is the minimum sample size calculated and it is mentioned in the methodology under “Sample size determination and sampling technique”.

6- In the analysis, make adjustment for the relevant, significant and confounding variables before concluding the results.

**Response:** Of course, there could be other confounding factors which could contribute for occurrence of malnutrition in patients with congenital heart disease. We have tried to avoid this effect by excluding those children with congenital heart disease and who are either syndromic or preterm or with chronic morbid conditions.

7- The authors include all types of congenital heart disease (I.e., simple defects and complex defect, treated/corrected and untreated/uncorrected). This point should be clear, revised, defended and properly discussed.

**Response:** In this particular study we have included those children with congenital heart disease for whom corrective intervention noy yet done. In a separate study which is partly a continuation of this study, we have tried to assess the impact of corrective surgery in those patients with congenital heart disease and malnutrition. We will send the result of this manuscript to the same journal in the near future. Indeed, some patients with congenital heart disease and malnutrition did not responded for the intervention which requires another explanation.
