# Supplementary material for: Nutritional assessment and associated factors in children with congenital heart disease—Ethiopia
Source: PLoS One. 2022 Sep 23;17(9):e0269518. doi: 10.1371/journal.pone.0269518 (PMC9506638; doi:10.1371/journal.pone.0269518)
Supplement: Attachment — Submitted filename: Review comments on PONE-D-21-07948_R1.docx [file pone.0269518.s003.docx]

Reviewer comments on PONE-D-21-07948_R1

I congratulate authors to prove that malnutrition status of 208 pediatric CHD patients in Ethiopian population generally agrees with the reported populations. Applying these results enables to consider dietary or nutrient strategies to certain risk careering CHD patients.

However, I have some critiques authors should address:

Informed consent

It is shown as “patient’s oral consent” in ethic statement of cover letter. However in the paper text, it is shown as “written consent”. Please harmonize.

Format and structure of the paper

The paper does not align with a standard journal paper format. For example, rather long introduction with short discussion. Authors should reconcile.

- Introduction sections 1.1 to 1.3 are repetitive. These sections should be consolidated into one section.
- Authors should remove section 2. literature review. Then, the contents can be integrated into discussion or introduction section.
- Section 3. Objectives. Authors should not use bullet style format.
- Section 4.1. Irrelevant information about hospital. Please reconcile.
- Sections 4.2-4.4: Repetitive, please consolidate
- Section 4.10. Irrelevant, please remove

Abstract

- Children age between 5-10? Since Authors revised table. Same applies to conclusion, please update.

Methodology

Section 4.4. Authors need citation of a research from Egypt to determine sample size.

Results.

- Authors conducted bivariate analysis for certain set of variables followed by multivariate analysis. In the table 5.x, authors showed only three variables each without mentioning other variables. Did other variables meet statistically significant associations? Authors should clarify this in the text.
- For bivariate analysis, authors used both Acyanotic CHD and cyanotic CHD. I do not think there were control population. Does this have to be “CHD type”?

Discussion

- In your population or general CHD studies, Do patients who have PAH exhibit more severe condition? Do patients only have ASD exhibit less severe condition? Then, it makes sense for the associations with malnutrition. At least, you want to include in the discussion.
