# Supplementary material for: Nutritional assessment and associated factors in children with congenital heart disease—Ethiopia
Source: PLoS One. 2022 Sep 23;17(9):e0269518. doi: 10.1371/journal.pone.0269518 (PMC9506638; doi:10.1371/journal.pone.0269518)
Supplement: Attachment — Submitted filename: RESPONSE to Reviewers.docx [file pone.0269518.s004.docx]

**Response to Reviewers**

We would like to extend our gratitude for the reviewers and editors for your time and valuable comments. We have addressed all the comments in three pages and the response is written below each comment accordingly. All revised contents in the new manuscript are highlighted with light blue color.

From one of the reviewers (comment #5) there was also a comment about the use of standard English. While revising the manuscript, we have tried to address some of the errors. We highlighted the corrected part in yellow.

Thanks

Temesgen

**Reviewer #2:**

The manuscript has been improved. We still have two comments:
1- In the discussion before the limitations section, the authors should highlight the clinical implications of the current study. These clinical implications should be clear and practical.
2- The authors should revise carefully the care checklist of the journal.
**Response:**

1- As a last paragraph in the discussion (just above “limitation of the study”) we have added a new paragraph which describes the clinical implication of our study.
2- The manuscript is **restructured** as per PLOS ONE ‘manuscript organization’ guideline. So, we made corrections on the first page and the table of contents as some contents need to be either reshuffled or removed. The correction made in the manuscript are highlighted in light blue color.

- Acronym and abbreviations - rewritten.
- The abstract page reduced to one.
- Abbreviations are removed from the abstract.
- According to manuscript organization, the literature review and objective are removed while the objective in the abstract part has been left as it was.
- Acknowledgment added.

**Reviewer #3:**

Informed consent
It is shown as “patient’s oral consent” in ethic statement of cover letter. However in the paper text, it is shown as “written consent”. Please harmonize.

**Response:**

In the abstract there is a phrase stating about consent and it is corrected and rephrased same as the one mentioned in the ‘ethical consideration’ as ‘**Data was collected from patient card and care givers of the children included in the study after obtaining their informed written consent using data inquiry sheet.’**

I could not find a phrase stating about consent in the cover letter, which I have attached again.

**Reviewer #3:**

Format and structure of the paper
The paper does not align with a standard journal paper format. For example, rather long introduction with short discussion. Authors should reconcile.
• Introduction sections 1.1 to 1.3 are repetitive. These sections should be consolidated into one section.

**Response:** We have minimized the introduction part by avoiding repetitive information.

- Section 1.2 and 1.3 are minimized and specially 1.3 is rewritten again.

**Reviewer #3:**

Format and structure of the paper Authors should remove section 2. literature review. Then, the contents can be integrated into discussion or introduction section.

**Response:** Literature review part is removed, and it was not even in the PLOS ONE journal manuscript organization format.

• Section 3. Objectives. Authors should not use bullet style format.

**Response**: According to manuscript organization the objective is removed while it is left in the abstract part as it was.

• Section 4.1. Irrelevant information about hospital. Please reconcile.

**Response**: Some unnecessary phrases are removed.

• Sections 4.2-4.4: Repetitive, please consolidate

**Response**: Re-written

• Section 4.10. Irrelevant, please remove

**Response:** removed

Abstract
• Children age between 5-10? Since Authors revised table. Same applies to conclusion, please update.

**Response: corrected as per the comment.**

Methodology
Section 4.4. Authors need citation of a research from Egypt to determine sample size.

**Response:** **Reference # 13.** Hassan BA, Albanna EA, Morsy SM, Siam AG, Al Shafie MM, Elsaadany HF, Sherbiny HS, Shehab M, Grollmuss O. Nutritional Status in Children with Un-Operated Congenital Heart Disease: An Egyptian Center Experience. Front Pediatr. 2015 Jun 15;3:53. doi: 10.3389/fped.2015.00053. PMID: 26125014; PMCID: PMC4467172.

• Authors conducted bivariate analysis for certain set of variables followed by multivariate analysis. In the table 5.x, authors showed only three variables each without mentioning other variables. Did other variables meet statistically significant associations? Authors should clarify this in the text.

**Response:** Multivariable analysis was done to see if there is significant association of factors identified on bivariate analysis. We are mentioning here only those variables which showed associations. We put this statement in the result part above the table (highlighted in blue color).

• For bivariate analysis, authors used both Acyanotic CHD and cyanotic CHD. I do not think there were control population. Does this have to be “CHD type”?

**Response:** We used this quantitative analysis to compare two variables which are CHD types otherwise we don’t have a control group.

Discussion
• In your population or general CHD studies, Do patients who have PAH exhibit more severe condition? Do patients only have ASD exhibit less severe condition? Then, it makes sense for the associations with malnutrition. At least, you want to include in the discussion.

**Response: yes. Those patients with PAH are at higher risk of developing malnutrition, especially wasting and stunting as we mentioned it in the result Table 5b and 5c.**

**The other finding in this study is the decreased risk of being underweight and stunted (70% (95% CI: 0.1-0.9)) in children with ASD than those CHD without ASD.**

**As per your comment we put the above statements in the discussion as well (page 18 and 19)**
