# Supplementary material for: Nutritional assessment and associated factors in children with congenital heart disease—Ethiopia
Source: PLoS One. 2022 Sep 23;17(9):e0269518. doi: 10.1371/journal.pone.0269518 (PMC9506638; doi:10.1371/journal.pone.0269518)
Supplement: Attachment — Submitted filename: Response to reviewers.docx [file pone.0269518.s005.docx]

Responses to reviewers

Hello Dears,

Thank you again for your time and continuous support.

Responses for the specific comments are highlighted yellow in the revised manuscript. One of the comments from reviewers was the use of standard English. I have gone through the manuscript again and corrected many grammatical errors and words which are shaded with red.

**Review Comments to the Author**

Reviewer #3: Authors have partly addressed my comments. Authors further need to address the comments below.

Informed consent
Please align to written consent from oral consent in Ethics Statement, which is in 5th page of the PDF manuscript (PONE-D-21-07948_R2)

**Response:**

Corrected as ‘written consent’.

Introduction:
Page 7 from line 8-12, I do not understand the wording here. Please reword using standard English.

**Response:**

Addressed accordingly, is highlighted in yellow.

Results:
Authors categorize acyanotic and cyanotic as CHD type in Table 2. Later, authors state both acyanotic CHD and cyanotic CHD have significant associations by bivariate analysis (last line of P15 and two more in P16 and 17). Author confirmed no control group was included. Please remove both “acyanotic CHD” and “cyanotic CHD”, and replace to “CHD type”.

**Response:** Corrected as per the comment; in the revised manuscript page 16 and 17

Discussion:
Comment: P18, line 1-3, 11 and 20, Authors added parentheses and patient numbers. These are confusing with the citations. Please remove.

**Response:**

Removed as per the comment. page 14 and 15 highlighted with red.

The last sentence on page 6 is removed because it does not explain specifically why Infants and children are more likely to suffer from poor nutrition compared to adults.
